#  Epstein-Barr Virus Encephalitis: A Case Report 

**Published:** 2015

**Authors:** Somayh HASHEMIAN, Farah ASHRAFZADEH, Javad AKHONDIAN, Mehran BEIRAGHI TOOSI

**Affiliations:** 1. Pediatric Neurology Department, Ghaem hospital, Mashhad University of Medical Sciences, Mashhad, Iran

**Keywords:** Encephalitis, Basal ganglia, Epstein-Barr virus

## Abstract

Many neurologic manifestations of Epstein-Barr virus (EBV) infection have been documented, including encephalitis, aseptic meningitis, transverse myelitis, and Guillain-Barre syndrome. These manifestations can occur alone or coincidentally with the clinical picture of infectious mononucleosis. EBV encephalitis is rare and is indicated as a wide range of clinical manifestations. We report a 10-year-old girl presented with fever, gait disturbance, and bizarre behavior for one week. The results of the physical examination were unremarkable. The diagnosis of EBV encephalitis was made by changes in titers of EBV specific antibodies and MRI findings. A cranial MRI demonstrated abnormal high signal intensities in the basal ganglia and the striatal body, especially in the putamen and caudate nucleus. EBV infection should be considered when lesions are localized to the basal ganglia.

## Introduction

Primary Epstein-Barr virus (EBV) infections in children are common and frequently asymptomatic. EBV can lead to various central nervous system (CNS) complications include encephalitis, meningitis, cerebellitis, acute disseminated encephalomyelitis (ADEM), transverse myelitis, and radiculopathy (1). EBV encephalitis is rare in children but can have severe neurological complications. Encephalitis with EBV has outcomes that vary from complete recovery to death. These manifestations can occur alone or in the setting of infectious mononucleosis (IM). Establishing a diagnosis of EBV encephalitis is difficult and consequently molecular, serological, and imaging techniques should be used when investigating children with encephalitis (2). The incidence of neurological complications and CNS symptoms during EBV infection may be the only clinical manifestations of IM. It is evident that EBV infection must be considered in the diagnosis of various acute neurological diseases affecting children, even in the absence of other signs of IM. 

## Case report

A 10-year-old girl was admitted to our hospital with 4-day history of consciousness disturbance and decreased activity. She had a 10-day history of fever and bizarre behavior such as junk feeding and abusiveness on the 2nd day of illness. Then, she had decreased activity with a gate disturbance and urine incontinency as well as a suspected history of seizure.

On admission, she was lethargic and an examination revealed normal vital signs. There was no lymphadenopathy or hepatosplenomegaly. In the neurological examinations, she was drowsy and unresponsive to verbal commands. Her pupils were normal sized and reactive to light. Other examinations were unremarkable except for an unsteady gait and neck stiffness. 

The laboratory findings included a leukocyte count of 5900/μl (segmental neutrophil 80%, lymphocyte 18%, and monocyte 2%), hemoglobin 12.8 gr/dl, platelet count 264000/μl, serum potassium 4.4 mEq/dl, sodium 141 mEq/dl, total calcium 9.5 mg/dl, urea 28 mg/dl, creatinine 0.6 mg/dl, glucose 132 mg/dl, cerebrospinal fluid(CSF) analysis revealed white blood cell 200/ mm3( lymphocyte 35 %, neutrophil 65%), red blood cell 100/mm3, protein 18 mg/dl, and sugar 89 mg/dl. 

The serum PCR was negative for herpes simplex virus 1, 2 (HSV 1, 2). Blood culture, urine culture, and CSF culture were all negative as well as a negative PPD test. An electroencephalogram (EEG) showed generalized slow waves. In brain CT scan, we found nonspecific evidence of brain edema. 

**Fig 1:**
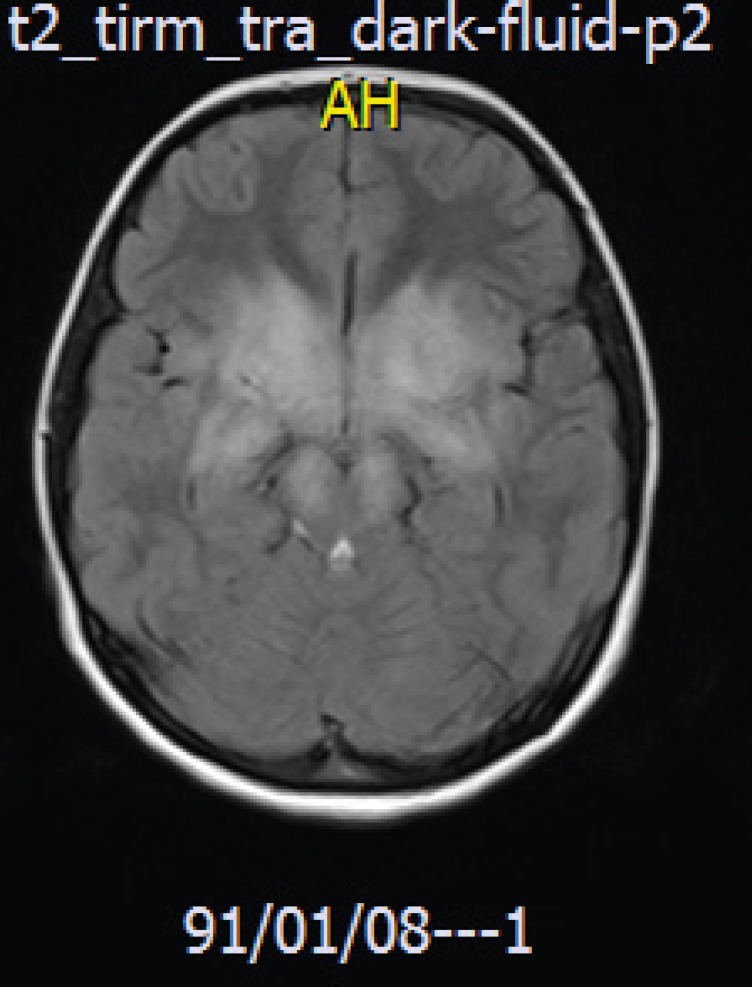
Brain MRI Flaire image showing hyperintensities in basal ganglias

**Fig 2:**
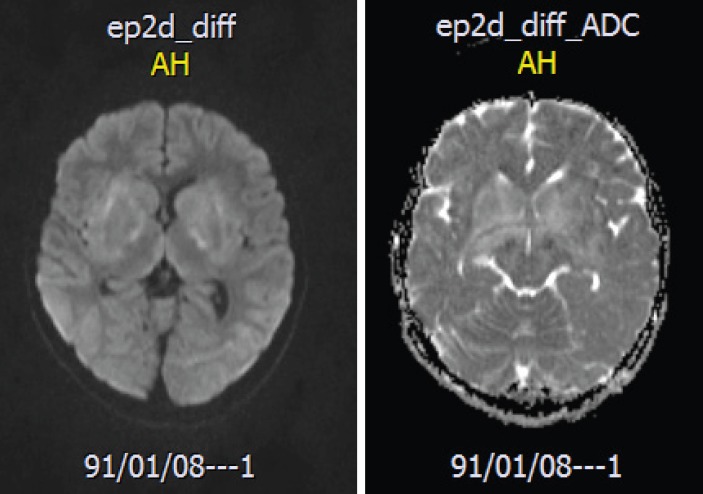
Brain MRI image DWI and ADC map showing no restricted

A brain MRI revealed high signal intensities in the basal ganglia in FLAIR image (figure1) and there was no restrictions in the lesion in the diffusion-weighted image (DWI) and the apparent diffusion coefficient map image (ADC-MAP), which ruled out vascular problems or stroke in our case (figure 2-**3**). With the brain MRI findings, we thought about EBV encephalitis and checked the serologic tests. Serologic testing was compatible with acute EBV infection, positive for viral capsid antigen (VCA) IgM and negative for VCA IgG. Encephalitis was confirmed by clinical and radiological findings. 

Empirical intravenous antibiotics and Acyclovir were administered, but stopped after the final negative CSF results for microorganisms were obtained. She gradually recovered and discharged after the 14th day of admission. At 6 weeks after the onset of illness, she could walk and speak well but could not go back to school. 

## Discussion

EBV is a well-known pathogen for infectious mononucleosis (IM). EBV infections have various manifestations alone or accompanied by clinical features of IM, such as meningo encephalitis, encephalitis, seizure, peripheral neuritis, Guillain Barre Syndrome, Bell’s palsy, and cerebellar ataxia (3,4,5). In 1931, the first reports of neurological complications in IM were described (6). 

Neurological complications of EBV infection occur in 1–18% of patients with infectious mononucleosis (7), according to previous studies on pediatric, EBV associated encephalitis. EBV infection was demonstrated in 2–9.7 % of children admitted with encephalitis (8). EBV infections of the CNS can occur in the absence of IM (5). 

Doja et al. reported all cases of EBV associated encephalitis compiled from 1994 to 2003. A total of 21 (6%) of 216 children with a median age of 13 (ranged 3–17) in the encephalitis registry were identified as having evidence of EBV infection. One patient had symptoms of classic IM and all others had a nonspecific signs, including fever (18%) and headache (66%). Slightly less than half (48%) had seizures and often had EEGS showing a slow background (57%); and 71% had abnormal MRI findings (9). 

There are a few reports describing detailed neurological examinations of EBV encephalitis. Kou et al. reported a girl who showed clinical signs of encephalitis during an acute infection with EBV, her brain MRI showed low and high intensity of both basal ganglia (predominantly in the putamen) on T1-w and T2-w images; while the brain CT scan demonstrated only mild edema (10). These MRI changes were similar to Ono et al. (6). Other studies have reported nonspecific findings with MRIs (7,9). Normal CT scans from these two reports suggested that the demyelinating process or vasculitis might play an important role in the basal ganglia lesions (6). 

Since EBV has a tropism for the deep nuclei, neuroimaging can show characteristic multiple foci of T2-weighted or FLAIR hyper intensity in the hemispheric cortex, brain stem, bilateral thalami, and basal ganglia. Rarely extensive white matter lesions have been reported in patients with chronic EBV infection and clinical relapse of neurologic problems (8).

In our patient, brain MRI showed high signal intensities in both basal ganglia. Brain CT scan had nonspecific brain edema. These findings are similar to Kou et al. (10) and Ono et al. (6). 

Disorders that affect both basal ganglia include Leigh syndrome, mitochondrial encephalopathy, Wilson disease, and glutaric aciduria type 2 (6). In our patients, these disorders were excluded by clinical, serologic, and immunologic tests. 

The prognosis associated with EBV encephalitis is controversial and usually associated with recovery in spite of the severe manifestations that may require assisted ventilation (6). EBV should be considered in any acute illness of uncertain etiology in the pediatric population (11). EBV infection should be considered when lesions are localized to the basal ganglia (6). 

**In conclusion**, EBV infection is a common identifiable cause of acute childhood encephalitis and remains the most common agent mimicking herpes simplex virus encephalitis that can be identified with MRI findings of high signal intensities in basal ganglia and documented with serologic tests.
